# Comparison of changes in lipid profiles of premenopausal women with early-stage breast cancer treated with different endocrine therapies

**DOI:** 10.1038/s41598-022-27008-x

**Published:** 2022-12-31

**Authors:** Kaiyue Wang, Lu Shen, Wei Tian, Suzhan Zhang

**Affiliations:** 1 Department of Breast Surgery, the Second Affiliated Hospital, School of Medicine, Zhejiang University, Hangzhou, 310009 China; 2 Cancer Institute (The Key Laboratory of Cancer Prevention and Intervention, China National Ministry of Education), Department of Surgical Oncology, the Second Affiliated Hospital, School of Medicine, Zhejiang University, Hangzhou, 310009 China

**Keywords:** Cancer, Health care, Oncology, Risk factors

## Abstract

Adjuvant endocrine therapy improves the prognosis of early breast cancer with hormone receptor positivity. However, there is no systematic report on the effect of endocrine therapy (particularly ovarian function suppression, OFS) on serum lipids in premenopausal women. This retrospective cohort study aimed to determine whether various endocrine treatments had different effects on blood lipids. This study enrolled 160 premenopausal patients with stage I–III breast cancer in eastern China. The initial diagnostic information was retrieved from patient's medical records, including age at the time of diagnosis, tumor characteristics, anticancer treatment and past medical history. The changes in blood lipids in patients receiving different types of endocrine therapy were compared at the 3rd, 6th, 12th, and 24th months after initiating endocrine therapy. Generalized linear mixed model was used in our analyses. Our data revealed that low-density lipoprotein cholesterol (LDL-C) levels in patients with tamoxifen (TAM) were significantly lower in the 6th, 12th, and 24th months than that in the 3rd month, while high-density lipoprotein cholesterol (HDL-C) levels in the 6th, 12th, and 24th months were significantly higher than that in the 3rd month, indicating that blood lipid levels generally improved with time. While in TAM plus OFS group, HDL-C levels were significantly higher in the 24th month than in the 3rd month, total cholesterol (TC) levels were significantly higher in the 24th month than in the 6th month. The lipid profiles of OFS plus aromatase inhibitor (AI) group did not show significant differences at any time point but were significantly higher than those of the other two groups especially in LDL and TC. TAM group tended to have lower serum lipid levels. With longer follow-up, no statistically significant difference in values was observed between TAM and TAM plus OFS groups at various time points. Compared with the other two groups, OFS plus AI group presented an increasing trend toward LDL-C and TC. The risk of dyslipidemia requires further investigation using a large sample size.

## Introduction

The International Agency for Research on Cancer (IARC) recently released the latest global cancer burden data for 2020^1^. Breast cancer (BC) now accounts for nearly 2.26 million new cases worldwide, surpassing lung cancer to become the most common cancer in the world. The incidence and mortality rate of breast cancer both rank the first in female malignant tumors in China^2^, and hormone receptor-positive (HR +) breast carcinoma is the most common subtype accounting for 60% of all breast cancers^3^. Adjuvant endocrine therapy should be used in patients with HR + BC to reduce the risk of disease recurrence and improve survival^4^. Endocrine therapy for premenopausal breast cancer patients, including tamoxifen (TAM), ovarian function suppression (OFS) and aromatase inhibitors (AIs), is based on lowering circulating estrogen levels. However, previous research indicates that estrogens possess a cardiovascular protective effect^5^. Reduced estrogen levels may result in dyslipidemia, which increases the risk of cardiovascular disease, and atherosclerotic cardiovascular disease (CVD) incidence increases rapidly concurrently^6^. With the increasing survival after early breast cancer, it is critical to pay adequate attention to chronic diseases such as dyslipidemia and cardiovascular disease^7^. Cardiovascular disease-related death has become the second most common cause of death in BC patients, particularly in postmenopausal women^8^. Some postmenopausal HR + BC patients receive aromatase inhibitors as endocrine therapy^9^. Numerous studies have demonstrated an increased risk of heart failure and cardiovascular events through elevated serum lipids in postmenopausal breast cancer women treated with aromatase inhibitors^10^. There is evidence that hypercholesterolemia during AIs treatment may impair the desired outcome of AIs^11^. Also, the primary metabolite of cholesterol can regulate estrogen receptor activity^12^. However, no large-scale study has been conducted on the impact of endocrine therapy (especially OFS) on blood lipids in premenopausal BC patients^13^. This study retrospectively analyzed lipid profiles of premenopausal BC patients who received different endocrine therapies in eastern China.

## Material and methods

### Study participants and design

We retrospectively analyzed premenopausal women with early-stage breast cancer who started endocrine therapy in the Second Affiliated Hospital of Zhejiang University from January 1, 2013, to December 31, 2017. Eligible patients were premenopausal, had hormone receptor-positive early-stage breast cancer, and received TAM,  OFS with TAM or OFS with an aromatase inhibitor (AI) as adjuvant endocrine therapy. Exclusion criteria included patients who suffered from another malignancy or tumor recurrence^14^, patients with dyslipidemia, certain cardiovascular disease (coronary artery disease, stroke, and hypertensive heart disease), diabetes mellitus combined with target organ damage, chronic kidney disease, smokers, and patients taking lipid-altering drugs. Patients were excluded from this lipid analysis if the lipid profile data were unavailable for any time points, including 3, 6, 12, and 24 months after initiating endocrine treatment.

Patients were stratified based on which type of endocrine therapy they received during a 2-year follow-up, including TAM, OFS plus TAM, and OFS plus AI. For each patient at baseline, we obtained medication history, disease history, physical examinations, laboratory test results, pathological results, and immunohistochemistry results (Table 1). We then collected blood lipid indexes tested in the Second Affiliated Hospital of Zhejiang University. Following that, we analyzed the levels of total cholesterol (TC), high-density lipoprotein cholesterol (HDL-C), low-density lipoprotein cholesterol (LDL-C), and triglyceride (TG) at the four times. Researchers recommended that participants with sharply elevated blood lipid levels seek clinical consultation from the cardiovascular department during follow-up (3, 6, 12, and 24 months after initiating endocrine treatment).

**Table 1:** Patient and treatment characteristics.

Characteristics	TAM (N = 112)	TAM + OFS (N = 37)	OFS + AI (N = 11)
	N (%)	N (%)	N (%)
Age (Median [IQR])	43[41–47]	38[34–42]	40[37–46]
BMI (Mean ± SD)	21.95 ± 2.56	22.15 ± 2.72	21.52 ± 2.97
**Surgery**			
Conserving	56 (50)	14 (37.84)	5 (45.45)
Mastectomy	56 (50)	23 (62.16)	6 (54.55)
**T (tumor)**			
T1	81 (72.32)	24 (64.86)	9 (81.82)
T2	25 (22.32)	13 (35.14)	1 (9.09)
T3	3 (2.68)	0	1 (9.09)
Unknown	3 (2.68)	0	0
**N (node)**			
N0	78 (69.64)	17(45.95)	9 (81.82)
N1	31 (27.68)	19(51.35)	2 (18.18)
N2	0	1(2.70)	0
Unknown	3(2.68)	0	0
**HER2**			
+	88(78.57)	33(89.19)	10 (90.91)
−	24(21.43)	4(10.81)	1 (9.09)
**Radiotherapy**			
Yes	72(64.29)	25(67.57)	10 (90.91)
No	40(35.71)	12(32.43)	1 (9.09)
**Chemotherapy**			
Yes	97(86.61)	37(100)	10(90.91)
No	15(13.39)	0	1 (9.09)

### Methods

Direct methods were employed to measure LDL-C and HDL-C levels. Serum TG was detected using glycerol-phosphoric acid oxidase peroxidase method, and TC was detected using cholesterol oxidase method. We analyzed the time point of the third month after starting endocrine therapy, and no significant difference (*P* > 0.05) was observed in the blood lipid profile among the groups. Accordingly, we take the blood lipid at the third month as the baseline to explore long-term change trend.

### Statistical analyse

Basic descriptive statistics, including mean and standard deviation (SD), were utilized to characterize study participants.Generalized Linear Mixed Model (GLMM) and Least Significant Difference (LSD) were used to compare lipid level variables with different endocrine therapies at varying time points. Statistical analyses were performed using SPSS 25.0 for Windows. *P* < 0.05 was considered statistically significant.

### Ethics approval

The institutional review board of the Second Affiliated Hospital of Zhejiang University approved the study. Informed consent was waived by the the Second Affiliated Hospital Zhejiang University School of Medicine human research ethics committee. All procedures performed in studies involving human participants were in accordance with the 1964 Declaration of Helsinki and its later amendments or comparable ethical standards.

## Results

### Study population

**Figure 1:**
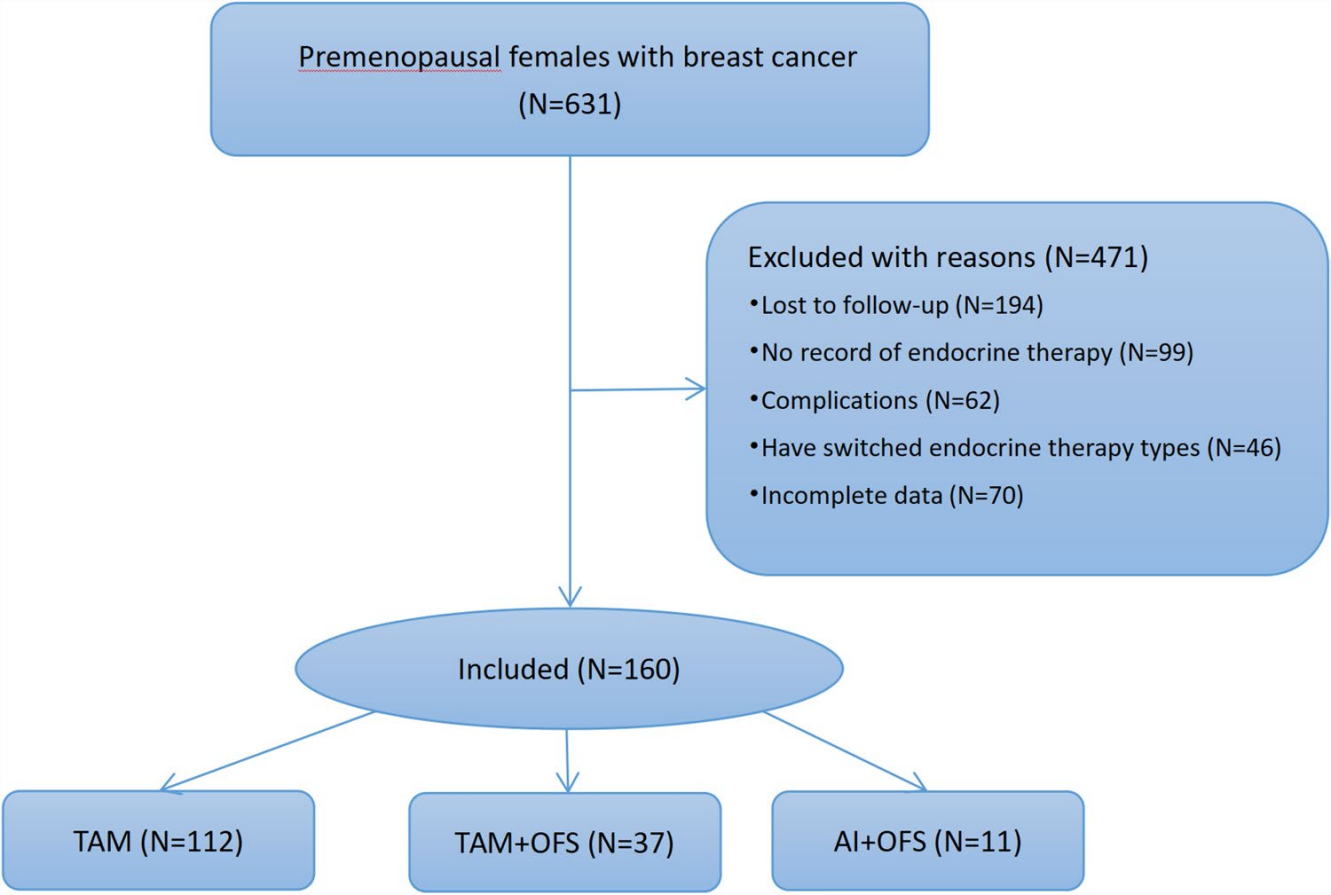
Review the process of grouping.

We collected individual data of 631 premenopausal females with breast cancer in the study, 160 were finally included (Fig. 1). The baseline characteristics for individual TAM (n = 112), OFS plus TAM (n = 37), and OFS plus AI (n = 11) groups are presented in Table 1. 5-year tamoxifen administration was the first recommendation for adjuvant endocrine treatment of early breast cancer, implying the high number of people in the TAM group.

### Lipid profiles

For these three different endocrine treatments, the average and standard deviation of blood lipid spectrum are displayed in Table 2. During endocrine therapy, the overall TC at the 24th month was significantly higher than that at the other three time points (*P* < 0.05), and there was no significant difference in TG and LDL-C among each time point. LDL-C values of OFS plus AI group were significantly higher than those of TAM plus OFS (*P* = 0.009) and TAM groups (*P* = 0.007) as a whole.

**Table 2:** Changes in lipid levels (mean and SD) from the third month to each time point among patients with different endocrine therapies.

	TAM				OFS + TAM				OFS + AI			
	3 months	6 months	12 months	24 months	3 months	6 months	12 months	24 months	3 months	6 months	12 months	24 months
	(baseline)				(baseline)				(baseline)			
TC	4.60 ± 1.11	4.50 ± 1.00	4.53 ± 1.07	4.54 ± 1.01	4.55 ± 0.89	4.39 ± 0.80	4.45 ± 0.76	4.60 ± 0.86	4.88 ± 1.23	4.91 ± 0.88	5.10 ± 1.14	5.36 ± 1.15
(mmol/L)		*P* = 0.088	*P* = 0.194	*P* = 0.308		*P* = 0.171	*P* = 0.416	*P* = 0.560		*P* = 0.866	*P* = 0.226	*P* = 0.017
TG	1.88 ± 1.63	1.91 ± 1.34	1.89 ± 1.74	1.74 ± 1.54	2.10 ± 1.56	1.79 ± 0.74	1.88 ± 1.20	1.82 ± 0.76	1.32 ± 0.50	1.25 ± 0.57	1.29 ± 0.45	1.13 ± 0.35
(mmol/L)		*P* = 0.782	*P* = 0.971	*P* = 0.192		*P* = 0.091	*P* = 0.194	*P* = 0.105		*P* = 0.852	*P* = 0.927	*P* = 0.613
LDL-C	2.38 ± 0.92	2.25 ± 0.83	2.25 ± 0.88	2.19 ± 0.84	2.30 ± 0.71	2.22 ± 0.66	2.22 ± 0.62	2.23 ± 0.70	2.73 ± 0.89	2.88 ± 0.60	2.91 ± 0.81	3.01 ± 0.79
(mmol/L)		*P* = 0.004	*P* = 0.004	*P* < 0.001		*P* = 0.356	*P* = 0.342	*P* = 0.337		*P* = 0.319	*P* = 0.194	*P* = 0.067
HDL-C	1.38 ± 0.32	1.40 ± 0.34	1.43 ± 0.32	1.51 ± 0.34	1.42 ± 0.34	1.43 ± 0.31	1.47 ± 0.32	1.51 ± 0.30	1.35 ± 0.24	1.35 ± 0.29	1.41 ± 0.31	1.47 ± 0.24
(mmol/L)		*P* = 0.409	*P* = 0.018	*P* < 0.001		*P* = 0.767	*P* = 0.076	*P* = 0.015		*P* = 0.972	*P* = 0.360	*P* = 0.107

*P* value correspond to baseline (treated with endocrine therapy for 3 months).

### Lipid changes within groups

The average HDL-C value in TAM group at 24th months (1.51 mmol/L) was significantly higher than that at 3rd (1.38 mmol/L, *P* < 0.001), 6th (1.40 mmol/L, *P* < 0.001), and 12th months (1.43 mmol/L, *P* < 0.001), and HDL-C value at 12 months was significantly higher than that at the 3rd month (*P* = 0.018). The average HDL-C of TAM group exhibited a gradually increasing trend. The average HDL-C value in TAM plus OFS group at the 3rd month (1.42 mmol/L) was significantly lower than that at the 24th month (1.51 mmol/L, *P* = 0.015).

The TC value of TAM plus OFS group at the 24th month (4.60 mmol/L) was significantly higher than that at the 6th month (4.39 mmol/L, *P* = 0.043), and TC value of OFS plus AI group at the 24th (5.36 mmol/L) month was significantly higher than that at the 3rd (4.88 mmol/L, *P* = 0.014) and 6th months (4.91 mmol/L, *P* = 0.017).

**Figure 2:**
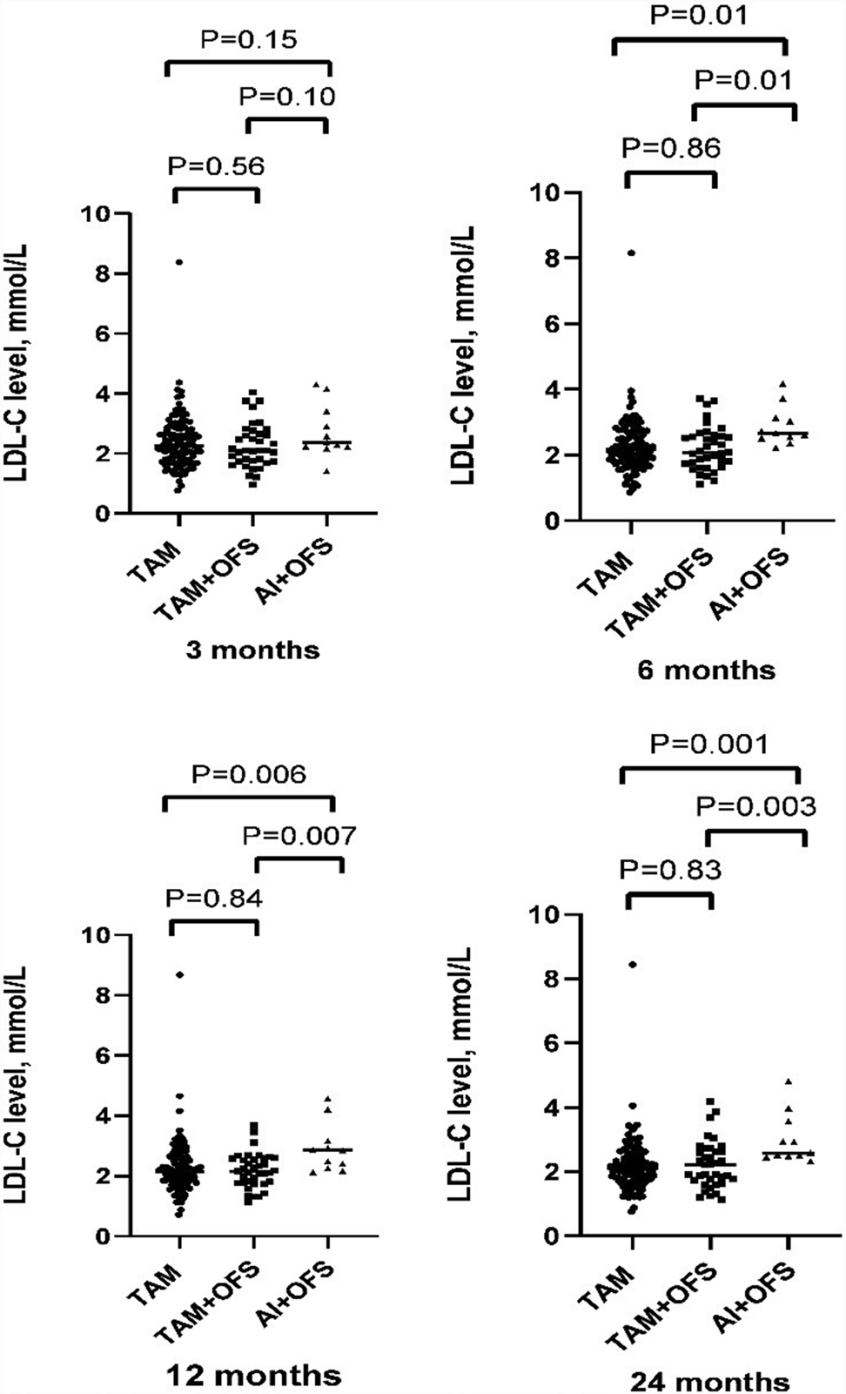
Low-density lipoprotein cholesterol (LDL-C) levels between different groups.

**Figure 3:**
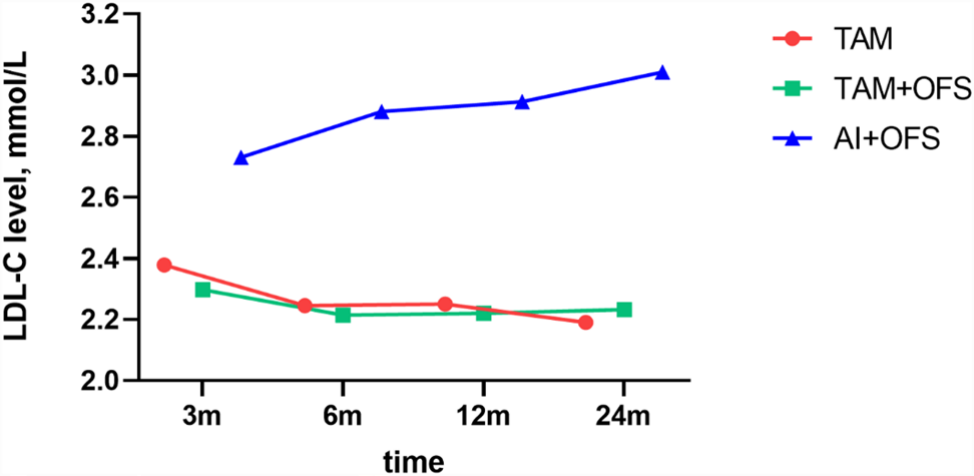
Low-density lipoprotein cholesterol (LDL-C) levels over time.

Average LDL-C values of OFS plus AI group demonstrated a gradually increasing trend, while LDL-C values of TAM group at the 3rd month were significantly higher than those at the later observation time points (*P* < 0.05) (Fig. 2). Different groups revealed an opposite trend (Fig. 3). LDL-C levels remained stable at all time points in OFS plus TAM group.

### Lipid changes between groups

The average TC value of OFS plus AI group at the 12th month was 5.10 mmol/L, significantly higher than that of TAM (4.53 mmol/L, *P* = 0.048) and TAM plus OFS (4.45 mmol/L, *P* = 0.041) groups at the same period. Similarly, TC value of OFS plus AI group at the 24th month was 5.36 mmol/L, significantly higher than that of TAM (4.54 mmol/L, *P* = 0.007) and TAM plus OFS (4.60 mmol/L, *P* = 0.019) groups.

The average LDL-C value of OFS plus AI group at the 6th month was 2.88 mmol/L, significantly higher than that of TAM (2.25 mmol/L, *P* = 0.010) and TAM plus OFS (2.22 mmol/L, *P* = 0.012) groups at the same period.The average LDL-C value of OFS plus AI group at the 12th month was 2.91 mmol/L, significantly higher than that of TAM (2.25 mmol/L, *P* = 0.006) and TAM plus OFS (2.22 mmol/L, *P* = 0.007) groups at the same period. The average LDL-C value of OFS plus AI group at the 24th month was 3.01 mmol/L, significantly higher than that of TAM (2.19 mmol/L, *P* = 0.001) and TAM plus OFS (2.23 mmol/L, *P* = 0.003) groups at the same period.

There was no significant difference in HDL-C between different endocrine treatment groups at each time point. In addition, no significant difference was observed in the absolute value of TG between the three groups at each evaluation point and among different regiments, but all fluctuated within a small range.

## Discussion

To prolong breast cancer survival, the comorbidities threat of breast cancer patients should be considered. There is a dearth of literature on the effect of different adjuvant endocrine therapies on lipid profiles in premenopausal breast cancer patients. The results of this study indicate that serum TC and LDL-C levels were significantly higher in patients treated with OFS plus AI than the other two groups (TAM group and TAM plus OFS group), implying a potential risk of dyslipidemia.

Tamoxifen has been the gold standard of adjuvant endocrine therapy for premenopausal BC patients with a relatively low risk of recurrence^15^. The finding is consistent with previous reports signifying that the overall blood lipid level improves over time with tamoxifen treatment. Numerous studies have demonstrated that TAM improves lipoprotein metabolism^16^. TAM use has been associated with decreased LDL-C and TC levels and an increased relative amount of HDL-C. TAM protects blood lipids during endocrine therapy, probably because its structure is similar to that of estrogen. It can compete with estradiol for estrogen receptors, form a stable complex with estrogen receptors, and perform an estrogenic function. Extensive evidence indicates that estrogen has a positive effect on blood lipids^17^.

No statistically significant difference was observed in blood lipid values between TAM and TAM plus OFS groups. This result is consistent with previous studies. A clinical trial has revealed no statistically significant differences in blood lipid levels between TAM-alone and goserelin plus TAM groups^18^. We conjecture that the positive effects of TAM compensated for the adverse effects of OFS. It is also possible that OFS does not have a significant negative effect on lipids.

Additionally, we are much more concerned with changes in lipid profiles in OFS plus AI group. During follow-up of long-term endocrine therapy (24 months), there were no significant differences in lipid profiles of OFS plus AI group at any time point, but LDL-C values exhibited an upward trend. A large-scale phase III prospective study SOFT trial reveals that 66% of premenopausal patients treated with exemestane plus triptorelin exhibited a profound, persistent reduction in E2 levels during the first 12 months of treatment, significantly greater than in tamoxifen plus triptorelin group at all time points^19^. This could be a contributing factor to dyslipidemia. When comparing our results to those of previous studies, it must be pointed out that AI may play a role in dyslipidemia. Research has indicated that compared with tamoxifen, AI use was linked to an increased cumulative risk of cardiovascular disease^20^. ITA and ACTC trials demonstrated that hypercholesterolemia was significantly more prevalent in anastrozole group over tamoxifen group among postmenopausal patients^21^, although the latter did not indicate an increased incidence of cardiovascular events^22^. However, no statistically significant difference was observed in the rates of cardiovascular events between letrozole and placebo groups, and no drug-related hypercholesterolemia was reported^23^. Premenopausal women will not benefit from these clinical trial findings because their hormone levels are higher than postmenopausal women^24^. Using AIs could theoretically increase the risk of dyslipidemia in premenopausal women whose hormones have reached menopausal levels following OFS therapy.

Although these studies reveal important discoveries, there are also limitations that we cannot determine whether OFS or AI has a negative impact on blood lipids in premenopausal women. The “baseline” differences in lipid levels were near significance. What is more, we applied strict criteria for inclusion and exclusion which may cause selection biases. We did not collect the data such as adherence to endocrine therapy or health behaviors and they may affect the final observation. Despite its exploratory character, this study can clearly indicate that different endocrine regimens may affect blood lipids.

There was a prospective clinical study comparing effects with exemestane plus ovarian suppression versus tamoxifen plus ovarian suppression, and dyslipidemia or CVD was not listed in adverse events of grade 3 or 4^25^. However, if a patient possesses a high risk of cardiovascular diseases, such as smoking, hypertension, hyperglycemia^26^, and obesity, clinicians may consider TAM an endocrine therapy when the risk of recurrence is similar. Estrogen receptor genotyping may assist in predicting whether premenopausal women would benefit more from tamoxifen^27^. When BC patients experience significant weight gain or are diagnosed with abnormal lipid metabolism during adjuvant endocrine treatment, short-term interventions such as behavior improvement (diet/exercise) can benefit BMI and blood lipids^28^. Even using long-term statin can improve overall survival and disease-free survival.

We can continue our investigation by considering the following aspects. First, the number of enrolled patients in the OFS plus AI group was small, influencing the statistical conclusions. If there are enough cases, comparisons between OFS and different AI can be conducted; however, different AIs may have varying effects on blood lipids^29^ and other safety concerns^30^. Steroidal and non-steroidal AIs differ in chemical structure and mechanism, affecting blood lipids^31^. Second, prospective randomized controlled trials are more convincing^32^, as they can control variables for factors affecting blood lipid such as diet and body weight, as well as to detect indicators such as sex hormones and apolipoprotein. Third, the findings can be further verified by animal experiments. Last, endocrine therapy usually lasts for five or 10 years, and we will continue to follow up for other complications such as coronary heart disease. Overall, research results will hopefully serve as useful feedback for future advances in prevention of dyslipidemia in receptor hormone-positive premenopausal breast cancer patients.

## Conclusions

In conclusion, for premenopausal hormone receptor-positive BC patients, TAM endocrine therapy demonstrated significant short- and long-term protective effects on serum lipids, as evidenced by a gradual decrease in LDL-C levels and increased HDL-C levels with prolonged medication time. No statistically significant difference was observed in lipid profiles between follow-up time points with OFS plus TAM treatment. OFS plus AI group revealed significantly higher TC and LDL-C levels at almost all time points than the above two treatments, implying that the combined treatment had a detrimental effect and may increase the risk of cardiovascular events. Different endocrine drugs and standard combinations will affect serum lipids, providing a starting point for further investigation. Generally, long-term management and follow-up are necessary to improve survival^33^.

## Data Availability

Te datasets analyzed in this study are not publicly available. Please contact the corresponding author regarding any reasonable requests.
